# International registry of congenital porto-systemic shunts: a multi-centre, retrospective and prospective registry of neonates, children and adults with congenital porto-systemic shunts

**DOI:** 10.1186/s13023-022-02412-8

**Published:** 2022-07-19

**Authors:** Simona Korff, Khaled Mostaguir, Maurice Beghetti, Lorenzo D’Antiga, Dominique Debray, Stéphanie Franchi-Abella, Emmanuel Gonzales, Florent Guerin, Anne-Lise Hachulla, Virginie Lambert, Periklis Makrythanasis, Nicolas Roduit, Laurent Savale, Marie-Victoire Senat, Joël Spaltenstein, Frank van Steenbeek, Barbara E. Wildhaber, Marcel Zwahlen, Valérie A. McLin

**Affiliations:** 1 Swiss Pediatric Liver Center, Department of Pediatrics, Gynecology, and Obstetrics, University Hospitals Geneva, University of Geneva, Geneva, Switzerland; 2 Clinical Research Centre, Geneva University Hospitals, University of Geneva, Geneva, Switzerland; 3 Congenital Heart Center, Division of Pediatric Subspecialities, Department of Pediatrics, Gynecology, and Obstetrics, University Hospitals Geneva, University of Geneva, Geneva, Switzerland; 4 Paediatric Hepatology, Gastroenterology and Transplantation, Hospital Papa Giovanni XXIII, Bergamo, Italy; 5 ERN RARE LIVER, Hamburg, Germany; 6 Pediatric Liver Unit, Competence Center for Rare Vascular Diseases, Necker Hospital, Assistance Publique Hôpitaux de Paris (AP-HP), Université de Paris, Paris, France; 7 Pediatric Radiology Department, Bicêtre Hospital, Assitance Publique Hôpitaux de Paris (AP-HP), Paris-Saclay University, Le Kremlin-Bicêtre, France; 8 Pediatric, Hepatology and Liver Transplantation, Reference Center for Liver Vascular Diseases, FSMR FILFOIE, Hépatinov, Inserm U 1193, Bicêtre Hospital, Assitance Publique Hôpitaux de Paris (AP-HP), Paris-Saclay University, Le Kremlin-Bicêtre, France; 9 Department of Paediatric Surgery, Bicêtre Hospital, Assitance Publique Hôpitaux de Paris (AP-HP), Paris-Saclay University, Le Kremlin-Bicêtre, France; 10 Division of Radiology, University Hospitals Geneva, Geneva, Switzerland; 11 Department of Paediatric Radiology, Bicêtre Hospital, Assitance Publique Hôpitaux de Paris (AP-HP), Paris-Saclay University, Le Kremlin-Bicêtre, France; 12 Congenital Cardiology Montsouris, Institut Mutualiste Montsouris, Paris, France; 13 Laboratory of Medical Genetics, Medical School, National and Kapodistrian University of Athens, Athens, Greece; 14 Department of Genetic Medicine and Development, Medical School, University of Geneva, Geneva, Switzerland; 15 Biomedical Research Foundation of the Academy of Athens, Athens, Greece; 16 Information Systems Department, University Hospitals Geneva, Geneva, Switzerland; 17 Faculty of Medecine, Paris-Saclay University, Le Kremlin-Bicêtre, France; 18 Department of Pulmonology and Respiratory Intensive Care, French National Reference Center for Pulmonary Hypertension, Bicêtre Hospital, Assitance Publique Hôpitaux de Paris (AP-HP), Le Kremlin-Bicêtre, France; 19 INSERM UMR_S 999, Marie Lannelongue Hospital, Le Plessis-Robinson, France; 20 Gynecology and Obstetrics Department, Bicêtre Hospital, Assitance Publique Hôpitaux de Paris (AP-HP), Paris-Saclay University, Paris, France; 21 OsiriX Foundation, Geneva, Switzerland; 22 Department of Clinical Sciences, Faculty of Veterinary Medicine, Utrecht University, Utrecht, The Netherlands; 23 Institute of Social and Preventive Medicine, University of Bern, Bern, Switzerland

**Keywords:** Congenital, Portosystemic, Shunt, Registry, Liver, Rare disease

## Abstract

**Background:**

Congenital portosystemic shunts (CPSS) are rare vascular malformations associated with the risk of life-threatening systemic conditions, which remain underdiagnosed and often are identified after considerable diagnostic delay. CPSS are characterized by multiple signs and symptoms, often masquerading as other conditions, progressing over time if the shunt remains patent. Which patients will benefit from shunt closure remains to be clarified, as does the timing and method of closure. In addition, the etiology and pathophysiology of CPSS are both unknowns. This rare disorder needs the strength of numbers to answer these questions, which is the purpose of the international registry of CPSS (IRCPSS).

**Method:**

A retrospective and prospective registry was designed using secuTrial® by the ISO certified Clinical Research Unit. Given that a significant number of cases entered in the registry are retrospective, participants have the opportunity to use a semi-structured minimal or complete data set to facilitate data entry. In addition, the design allows subjects to be entered into the IRCPSS according to clinically relevant events. Emphasis is on longitudinal follow-up of signs and symptoms, which is paramount to garner clinically relevant information to eventually orient patient management. The IRCPSS includes also three specific forms to capture essential radiological, surgical, and cardiopulmonary data as many times as relevant, which are completed by the specialists themselves. Finally, connecting the clinical data registry with a safe image repository, using state-of-the-art pseudonymization software, was another major focus of development. Data quality and stewardship is ensured by a steering committee. All centers participating in the IRCPSS have signed a memorandum of understanding and obtained their own ethical approval.

**Conclusion:**

Through state-of-the-art management of data and imaging, we have developed a practical, user-friendly, international registry to study CPSS in neonates, children, and adults. Via this multicenter and international effort, we will be ready to answer meaningful and urgent questions regarding the management of patients with CPSS, a condition often ridden with significant diagnostic delay contributing to a severe clinical course.

**Supplementary Information:**

The online version contains supplementary material available at 10.1186/s13023-022-02412-8.

## Background

Congenital portosystemic shunts (CPSS) are a rare malformation in humans accepted to result from incomplete vascular remodeling during embryogenesis, whereby the blood leaving the intestine does not pass entirely through the liver via the portal vein. Instead, it bypasses the liver partially or completely through an anatomically aberrant vessel connecting the porto-mesenteric system to the systemic venous circulation. CPSS are broadly divided into two categories: intrahepatic (IH) and extrahepatic (EH). The significance of this distinction is two-fold. First, they are anatomically different and therefore the relative proportion of portosystemic bypass differs between forms, with EH shunts often associated with a much more significant amount of splanchnic blood derivation than IH. Second, IH seem to be more likely to close spontaneously early in life [1, 2].

The systemic repercussions of abnormal portosystemic shunting and decreased hepatic portal flow are numerous and potentially serious. They include signs and symptoms such as hepatic encephalopathy, pulmonary vascular diseases (hepatopulmonary syndrome or pulmonary arterial hypertension), benign or malignant liver tumors, and biological and metabolic disorders [3–6]. Other less well-described associations include in utero growth retardation or tall stature in infancy, nephropathy, thyroid dysfunction, and coagulation abnormalities. In adults, CPSS have been reported at all ages, often as incidental findings, although in referral centers they are now sought as part of the work up of pulmonary hypertension [7].

The true incidence of CPSS is unknown, but it is estimated to affect 1/30–50,000 live births [4, 8]. CPSS is also known to be associated with other malformations or syndromes, such as congenital heart disease, heterotaxy, polysplenia syndrome, chromosomal anomalies, cutaneous or hepatic hemangiomas [3, 4, 9]. CPSS can be diagnosed at any age, either because the patient is symptomatic or as an incidental finding. Given their rarity, patients presenting with symptoms of systemic complications may experience significant diagnostic delay, because many of CPSS signs and symptoms mimic other conditions. Spontaneous closure of the CPSS has been reported to occur in some anatomic forms during the first one to two years of life [1, 2]. When the CPSS remains patent, radiologic or surgical closure of the CPSS may prevent, resolve, or stabilize complications [5, 6, 9, 10]. Yet, the conspicuous gap to fill in our current knowledge is which patient will benefit from shunt closure and when.

A preliminary analysis of 246 patients included 122 IH, 120 EH, and 3 patients with both IH and EH followed in centers participating in the IRCPSS [1, 2]. Among patients diagnosed pre-natally, a majority had IH shunts (75%). 189 (76%) patients were diagnosed post-natally at a mean age of 39.1 mo (0–200) for IH and 61.9 mo (0–192) for EH. IH and EH shunts were equally frequent when diagnosed after birth. IH shunts were more often an incidental finding. 24% of all CPSS were identified pre-natally. Among patients diagnosed post-natally, symptoms were equally frequent among patients with IH (57%) or EH (61%) CPSS. In addition, patients with EH CPSS were more likely to have several symptoms than patients with IH CPSS. They were also more likely to have liver nodules on imaging (40.7% vs. 26%). Closure: 184 children with CPSS were closed including 11 patients with 2 steps closure. Among these 184 patients 54% of IH CPSS and 5% of EH CPSS closed spontaneously. 46% of IH CPSS required medical or surgical closure of which nearly 40% for a preventive indication. 94% of patients with EH CPSS were closed through a procedure, of which 41% were preventive. Based on these preliminary data, it can be stated that IH and EH shunts were equally frequent in this multicenter retrospective cohort of CPSS in children. CPSS are a cause of severe symptoms in children and should be sought in infants with hypoglycemia or cholestasis. In older children, they should be considered in the differential diagnosis of liver nodules, cardiopulmonary symptoms or neurocognitive deficits. Given the potential severity of complications, preventative closure should be considered, although timing and approach need further study, something which the IRCPSS aims to address. While this preliminary analysis has offered some insight into the diagnosis and management of CPSS, much is unknown regarding both etiology and management, which is the basis for the registry presented herein.

Therefore, the main aim of the International Registry of Congenital Porto-Systemic Shunts (IRCPSS) is to characterize the clinical course of patients with CPSS and to identify subjects at risk of developing systemic complications with the overarching goal to stratify risks and standardize care of patients with CPSS. Although CPSS are increasingly suspected and sought in specialized centers, much is still unknown clinically, histologically, and biologically.

## Methods

### Study design

Given the rarity of CPSS, it is crucial to emphasize the importance of recording both prevalent and incident cases and to include as many centers as possible. It is for these reasons that the registry is designed as both a retrospective and prospective cohort study. At the time of print, it is estimated that > 500 retrospective cases are currently followed by centers participating in the IRCPSS. Although medical records may be incomplete, we aim to include all cases in the registry given the rarity of the condition and the importance of garnering sufficient numbers to improve the understanding of the full spectrum of these rare malformations.

#### Descriptive data

The following parameters are essential to improve our understanding of the medical course of patients with CPSS: medical history, anatomical characteristics of the CPSS, radiological progression of the CPSS over time, and the longitudinal follow up of signs and symptoms over time. Therefore the registry is designed to include clinical, imaging, histological, and biological data collected from the day of diagnosis until five years after CPSS closure (spontaneous or procedural), or any clinically relevant visit thereafter. As this is not an interventional trial, each participating center sets its own clinical follow-up schedule and enters into the registry what the site principal investigator (PI) deems to be clinically relevant visits.

#### Imaging

Imaging is crucial to diagnose and characterize CPSS, and to develop a clear and consensual nomenclature. Images are collected in all collaborating centers and stored in a dedicated imaging server meeting all current security norms. Scanned histology slides can be uploaded to the same server. Images are linked to the clinical registry using a secure process described below (image management).

### Study population

Since the registry aims to understand the full spectrum of CPSS, the IRCPSS includes neonates, children, and adults and follows subjects longitudinally. Any neonate, child or adult with CPSS is eligible for inclusion. Patients diagnosed in utero are enrolled at birth. Patients with chronic liver disease and secondary shunts are excluded.

### Model conception

Understanding the clinical question is central to developing the optimal registry. In the case of CPSS the crux of managing patients is to determine who will develop systemic complications, who will close spontaneously, and consequently who will need shunt closure, and when. While the preliminary analysis described above helps guide which patients warrant a closer follow-up, it is still unclear which patients may (not) need surgical or radiological closure, both procedures being laden with risks of significant complications, something which the registry aims to capture. Furthermore, it remains to be determined, among those patients who will develop associated signs or symptoms, which of these will regress following shunt closure, and which will need further specialized treatment. Therefore, the IRCPSS needs to capture longitudinal information in order to offer investigators the opportunity to analyze these very specific questions in nested studies.

There are several other challenging aspects of managing patients with CPSS which the registry aims to tackle. Among them, some of the primordial questions include how to proceed with complex shunts, how to follow up shunts diagnosed in utero*,* and the complexity of managing CPSS in the setting of congenital heart disease. The main clinical questions and goals of the IRCPSS determined the parameters (Table 1) as well as the structure of the registry (Table 2).

**Table 1:** List of parameters recorded in the IRCPSS

*Longitudinal data*
Signs and symptoms
Clinical exam
Imaging
Laboratory
Cardio-pulmonary complications
Adverse events and complications related to closure
*Crossectional data*
Inclusion criteria
Demographics
Medical history
Pre-natal history
Mode of presentation
Associated malformations (pre- and post-natal)
Shunt classification (pre- and post-natal)
Indication for closure
Type of closure and procedure
Available tissue samples and genetic workup
Termination of follow up

**Table 2:** Parameters recorded in the IRCPSS related to main clinical questions and goals

Clinical questions and goals	Cross-sectional data	Longitudinal data
Clear and consensual nomenclature	Shunt classification linked to images	
Correlation of prenatal findings with post-natal anatomy	Patient medical history, mode of presentation, associated malformations, shunt classification	
Type of cardiopulmonary complication and prognosis	Mode of presentation	Cardio-pulmonary complications
Who will develop associated signs or symptoms?	Pre-natal history, pas medical history, associated malformations, mode of presentation, shunt classification	Signs and symptoms before closure
Which signs or symptoms regress after shunt closure and which will need further specialized treatment		Signs and symptoms before and after closure
Closure, who and when? Surgical or radiological closure?	Associated malformations, shunt classification, mode of presentation, indication for closure, type of closure and procedure	Signs and symptoms, clinical exams, imaging, laboratory, adverse events and complications
How to proceed with complex shunts?	Associated malformations, shunt classification, mode of presentation, indication for closure, type of closure and procedure	Shunt identification, signs and symptoms, clinical exam, imaging, laboratory, adverse events and complications

### Clinical data collection

Only available data collected as part of routine clinical care of each center are recorded in the registry; additional medical visits or lab draws are not necessary for participation. Informed consent is obtained from participants or legal representatives in the case of pediatric subjects according to the ethical legislation of the country.

There are two semi-structured data sets. A Minimal Data Set (MDS) is available to include and to facilitate data entry for historical cases whose files are often very incomplete. The MDS does not include pre-natal data, medical history, pre-closure visits, or surgical or radiological information at closure. In turn, the Complete Data Set (CDS) is designed for prospective data and complete retrospective data. It includes pre-natal data and data listed in Table 1.

**Fig. 1:**
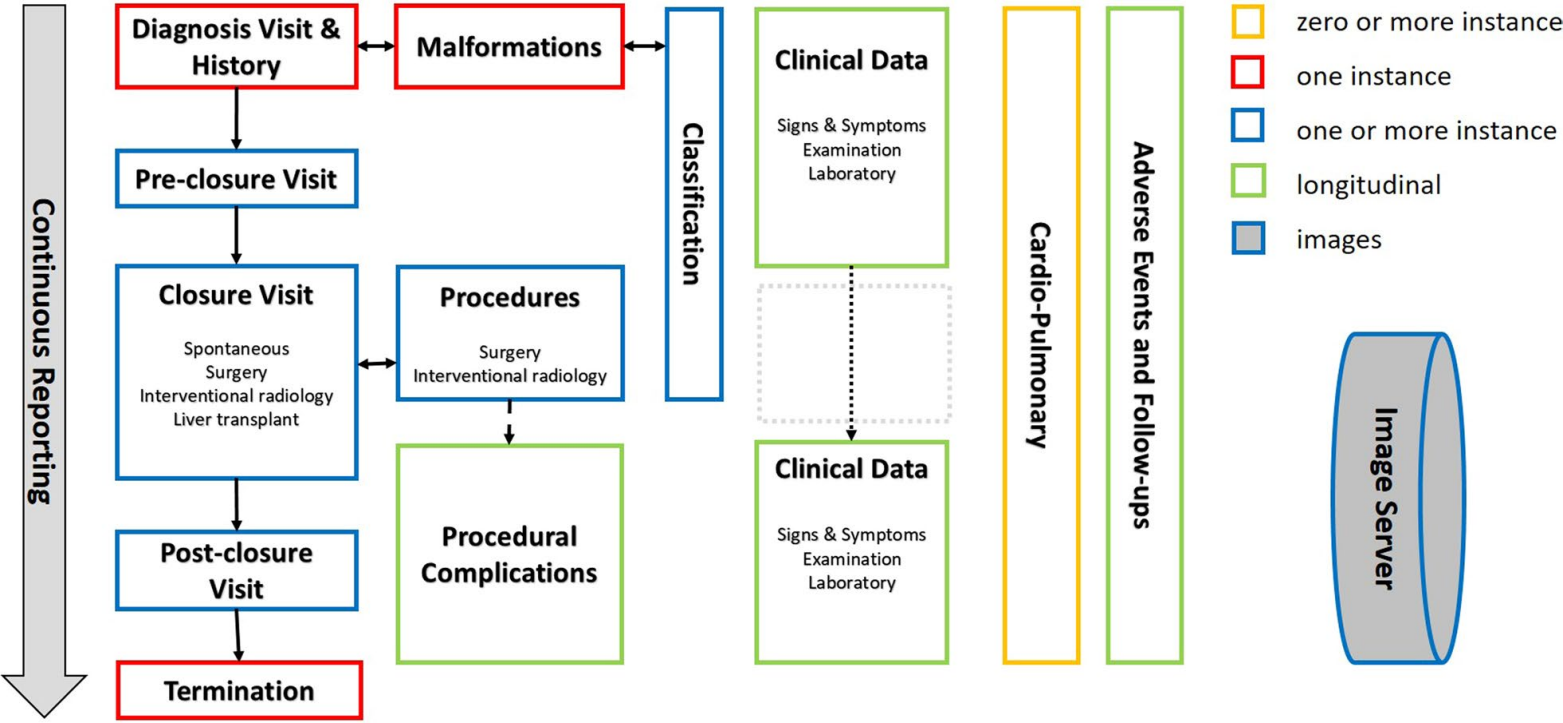
Structure and longitudinal collection of the complete data set

Data collected at each visit include demographic data (age, sex, weight, and height), clinical exams, and laboratories. Information on the type of imaging performed is also captured (angiography, magnetic resonance imaging, scintigraphy, ultrasound, computed tomography, electrocardiogram). Signs and symptoms are tracked at each visit in order to follow their course longitudinally before and after shunt closure (Fig. 1). For quantifiable data, such as liver nodules, it is possible to follow number and size over time.

Additionally, some data are collected at specific time-points during follow-up. At the first visit—also called “Basic Data & Early Diagnosis visit”—the following are recorded: patient medical history, shunt mode of presentation, and anatomical classification according to imaging [11]. In the registry each complex shunt is identified and has its own classification to facilitate follow-up through multiple closures. If a new shunt is identified, it can be managed independently from the first one. Associated malformations and prenatal data are also recorded by the system when available. Specific surgical and radiological data are collected during the closure visit. Cardio-pulmonary complications are documented and followed in a specific and independent cardio-pulmonary form which can be collected/recorded at any time-point. There are three [3] specific forms to be completed independently by specialists: cardiopulmonary, surgery, interventional radiology (Fig. 1). Given that clinically relevant cardiopulmonary visits may be required at different time points than shunt-specific visits, these can be entered independently. If the clinical course of the patient is favorable, follow up in the registry is terminated at five years after the last closure or at the last visit if the follow-up of the subject is shorter. A more detailed structure of the registry in UML presentation is available as Additional file 1.

### Data management and collection

Data management is performed by the Clinical Research Center at Geneva University Hospitals and Faculty of Medicine. The unit is certified ISO 9001/2008, and guarantees full compliance with Good Clinical Practices (GCP). Physical and electronic access to the data center is logged and limited to authorized personnel. Vulnerability testing is performed regularly to reduce potential exposure. The internal network is protected by multiple firewalls, proxy, reverse-proxy and state-of-the-art anti-virus solutions. Data are managed and physically stored using secuTrial® [12], a professional, specialized, browser-based, and GCP-compliant system for collecting, validating, and visualizing patient data in clinical research trials and registries. secuTrial® was adapted specifically to the complex needs of the registry. Data collected for each research subject constitute the subject’s electronic Case Report Form (eCRF). Each eCRF includes the parameters and follows the structure previously described under “Clinical Data Collection”. Research subjects are automatically pseudonymized and access to their data is strictly limited to authorized users. No personal data or data that may easily identify subjects are recorded, also for imaging. Each participating center has its own dedicated and protected area. The forms containing the data are edited and accessed by the study personnel based on a pre-defined set of roles and authorizations. All system logins, interventions, data modifications, and form status changes are thoroughly recorded for audit purposes.

#### Data quality

**Fig. 2:**
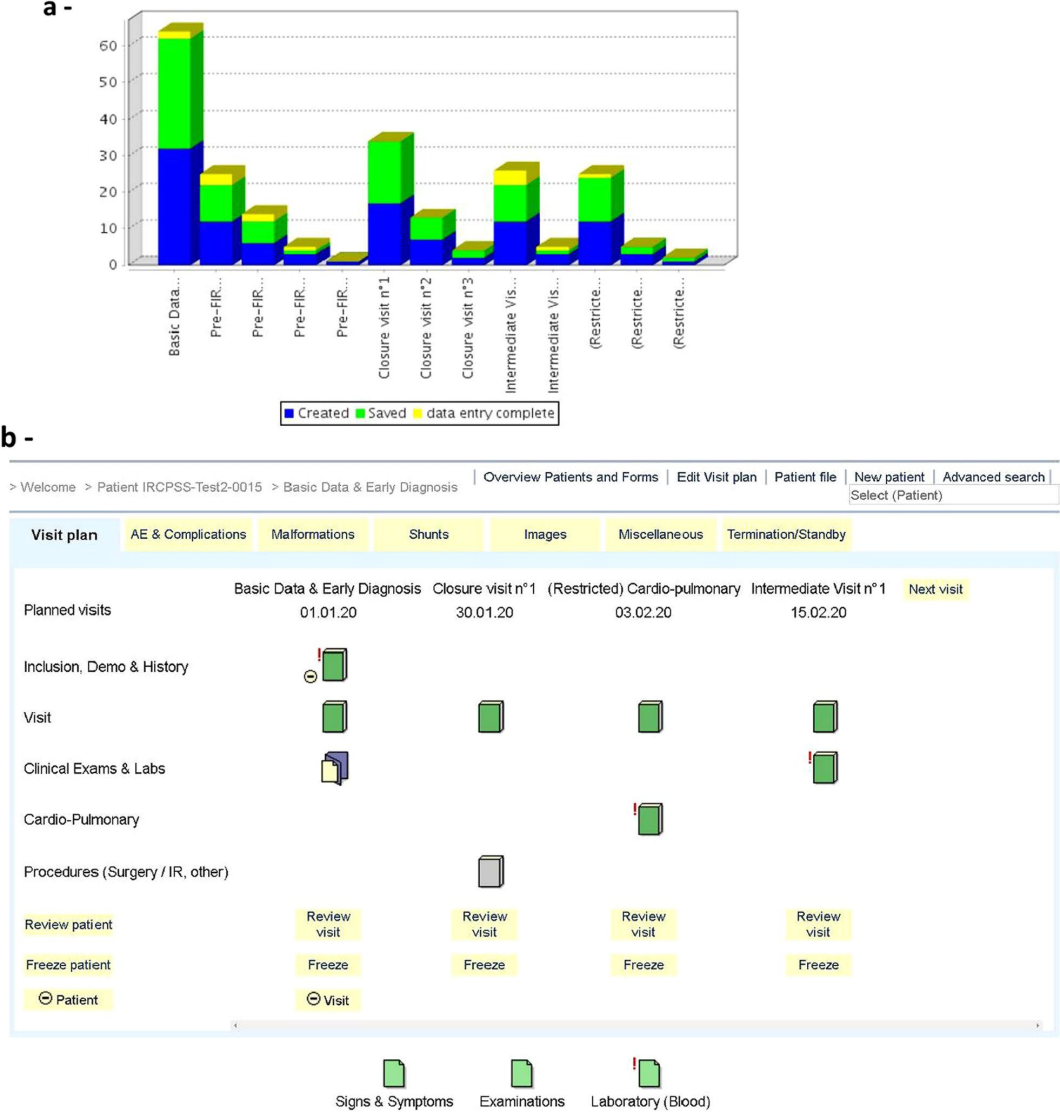
**a** secuTrial®: overview of the data entry progress and completion available in real time for each center. **b** secuTrial®: overview of subject visit plan and forms

The implementation of the data model was designed to avoid redundancy, to facilitate data capture, while minimizing logic and typing errors (e.g., automatic conversion of laboratory values, assessment of newly entered values with regard to previously entered ones, etc.). In addition to the automatic quality rules, data validation and review is thoroughly ensured by members of the registry steering committee (SC). To facilitate data and activity monitoring, up-to-date graphical and text reports, as well as descriptive statistics, are available in real time (Fig. 2a). Data management is further strengthened by an interactive, electronic process to comment, query and claim corrections, thereby ensuring high data quality.

#### Navigability

The built-in graphical interface has been optimized to facilitate navigability as much as possible during the data capture and data validation processes (Fig. 2b). Color codes indicate completion, validation, and accuracy status of the different data sections facilitating navigation between different visits and clinical categories (or sub-domains) by investigators.

### Image management

In addition to clinical data, the project includes DICOM imaging data that has been de-identified using the KARNAK [13] gateway. All personally identifying information (PII) is removed from non-pixeldata DICOM attributes. In cases such as ultrasound imaging, where PII is often burnt-in to the pixeldata, the images are modified to remove the identifying information. Image management is performed within a dedicated image server built on the KHEOPS platform [14]. The KHEOPS platform provides specific access controls based on users’ needs and an organizational structure, such as images submitted by different recruitment centers, remain isolated from each other. The image server is hosted in the data center of the Campus Biotech in Geneva under the responsibility of the Institute of Translational Molecular Imaging. This institute is affiliated with the University of Geneva and the Swiss Federal Institute of Technology. Within secuTrial®, each patient eCRF includes a redirection link that ensures a dynamic connection between the clinical registry and the image server.

### Data analysis

The planned statistical analyses are partly of a descriptive and partly of a comparative nature. Examples of descriptive analyses include describing the prevalence and incidence of a particular extrahepatic complication related to CPSS: for example pulmonary hypertension, liver nodules or tall stature. Each sign or symptom can in turn be studied by describing associations with neonatal findings or analyzing severity, treatment, and outcomes.

Comparative analyses will compare demographic or clinical characteristics between patients with or without a given sign or symptom: for example looking for differences between patients who develop pulmonary hypertension and those who do not. Among those who do, functional characteristics (for example New York Heart Association functional class) will be compared and regression models developed with the aim to identify associations.

### Description of recruiting centers

The first step for a new center to be part of the IRCPSS is to obtain the approval of its ethics committee. Consent forms are available in English, French, German, and Italian. If needed a Data Transfer Agreement (DTA) model has also been established by the legal department of University Hospitals Geneva to govern the transfer, use, and protection of data.

Given the complexity of CPSS management, it is expected that each collaborating center include a hepatologist, surgeon, radiologist, cardiologist, and pulmonologist on their CPSS team to enroll patients in the registry. The highly specialized nature of the data is beyond the expertise of most research coordinators, and therefore specialists have to enter highly specialized data. The site PI of each center acts as a coordinator among specialties adapting to the configuration and availability of local resources. Regardless, it is expected that cardiologists enter their data, given the complexity of the specific reports. For data quality issues, each specialist validates the data for his particular sub-specialty in his center, and a second level of validation is performed by the SC.

The prototypic participating center is a recognized, national and international center for liver disease with expertise in CPSS diagnosis and management in children or adults. At the time of print, 56 centers in 23 countries are part of the registry, 17 of which have obtained the approval of their ethics committee and 11 have initiated data entry.

### Data stewardship and publications

All centers participating in the IRCPSS have access to their own data and may agree to share their data with another center under the auspices of an ancillary project validated by the SC. Members of the SC have access to all data for oversight and quality control. Publication policy is defined by the SC in a Memorandum of Understanding signed by all centers entering data in the registry. Authorship is determined according to the scientific contribution of an individual to a given project or manuscript, and patient enrollment is acknowledged by listing all site contributors in alphabetical order in the ‘contributors’ section as found in PubMed. Partners wishing to conduct a nested study may submit to the SC a short study proposal for review and approval by the SC (contact via www.ircpss.com).

## Discussion and conclusion

The purpose of the IRCPSS registry is to bring together a vast collection of longitudinal, clinical, and imaging data across several systems in order to characterize the clinical course of patients with CPSS in detail with a view to harmonize clinical decision making and improve patient management. The challenge has been to build a solid and suitable data model encompassing the various clinical sub-domains and their complexity, while remaining as flexible as possible to facilitate data capture, ease of use, and long-term adaptability. We believe that we have successfully designed a tool that also offers the required flexibility to capture data across the many participating centers. It also offers the possibility for future technical evolution, including towards the development of interventional studies.

CPSS are a rare condition, and previous single-center studies have not been powered enough to answer some of the key questions that this large multicenter registry will be able to tackle. One of the unique features of this registry is that it brings together longitudinal data about the CPSS itself, with that of several organ systems in order to uniquely follow the course of systemic signs and symptoms over time. Furthermore, the creation of the IRCPSS has garnered the interest of multiple centers across continents. Importantly, it has succeeded in securing the commitment of multidisciplinary teams in all these centers. This is essential for developing a detailed understanding of CPSS and stems from the existent reality of clinical practice in the respective centers. In most instances, patients with CPSS cannot be managed in isolation, but rather need the collective know-how of multidisciplinary teams comprised of interventional radiologists, hepatologists, surgeons, endocrine specialists, pulmonologists and cardiologists, an aspect which this registry also aims to favor.

Finally, CPSS are complex vascular malformations with potentially severe systemic consequences often masquerading as other common complaints. Increasing awareness is therefore paramount and the creation of the IRCPSS enhance the visibility of this rare vascular malformation.

## Supplementary Information

**Additional file 1. Supplementary Figure**. UML presentation: package/class & use case diagram - relationships: 0..1 no instance or 1 instance, 0..* zero or more instance, 1..* at least one instance Purple: visits over time Yellow: clinical forms and imaging Green: specific forms at the Basic Data & Diagnosis visit Red: specific forms at closure Grey: Specific forms at any time-point.

## Data Availability

Not applicable.

## Abbreviations

CPSS: Congenital portosystemic shunts
IRCPSS: International Registry of Congenital PortoSystemic Shunts
IH: Intrahepatic
EH: Extrahepatic
MDS: Minimal Data Set
CDS: Complete Data Set
GCP: Good Clinical Practices
eCRF: Electronic Case Report Form
SC: Steering committee
ITMI: Institute of Translational Molecular Imaging
EPFL: Swiss Federal Institute of Technology
DTA: Data Transfer Agreement
PI: Principal Investigator
