# Supplementary material for: International registry of congenital porto-systemic shunts: a multi-centre, retrospective and prospective registry of neonates, children and adults with congenital porto-systemic shunts
Source: Orphanet J Rare Dis. 2022 Jul 19;17:284. doi: 10.1186/s13023-022-02412-8 (PMC9295381; doi:10.1186/s13023-022-02412-8)
Supplement: Supplementary file 1 — Additional file 1. Supplementary Figure. UML presentation: package/class & use case diagram - relationships: 0..1 no instance or 1 instance, 0..* zero or more instance, 1..* at least one instance Purple: visits over time Yellow: clinical forms and imaging Green: specific forms at the Basic Data & Diagnosis visit Red: specific forms at closure Grey: Specific forms at any time-point. [file 13023_2022_2412_MOESM1_ESM.pdf]

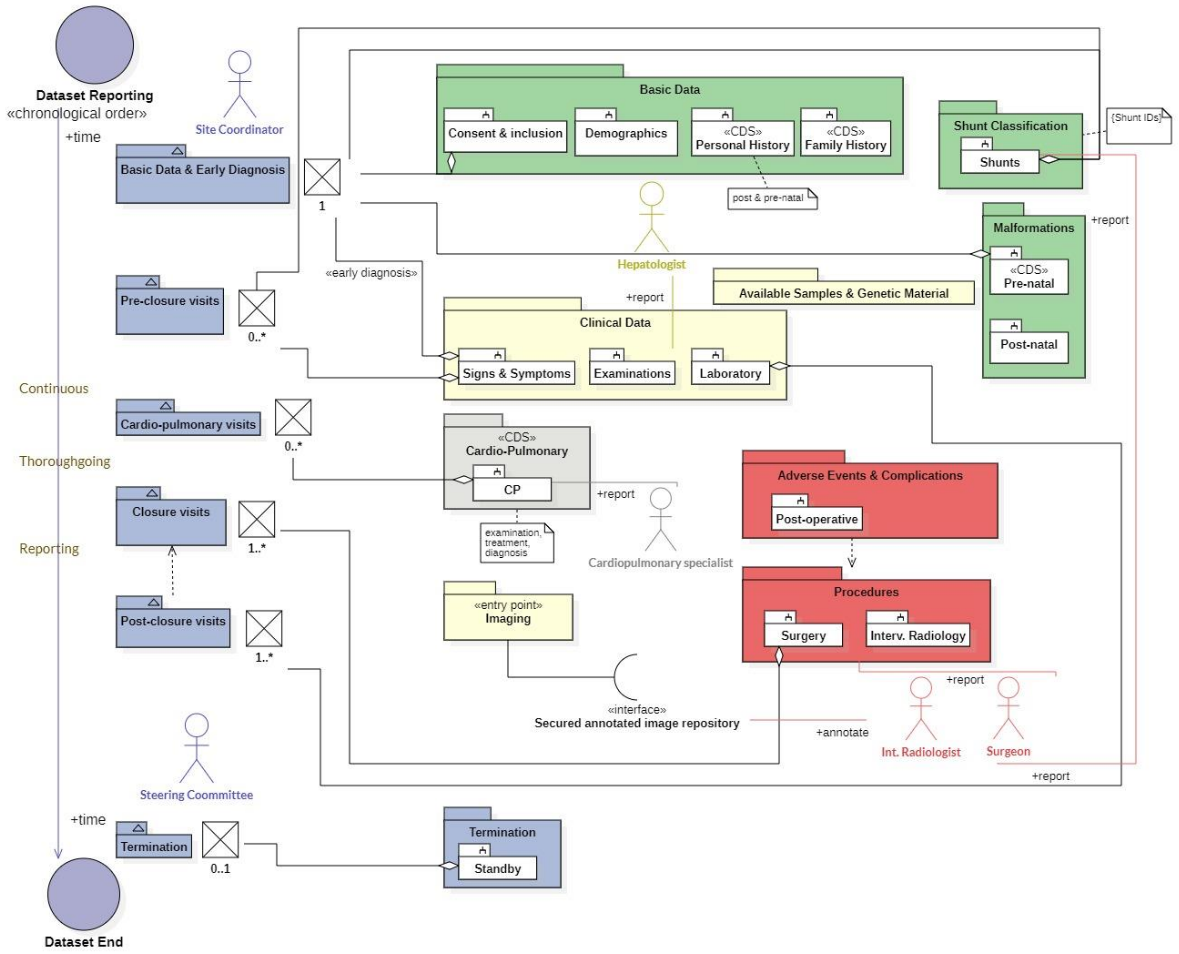

Supplementary Figure - UML presentation: package/class & use case diagram - relationships: 0..1 no instance or 1 instance, 0..* zero or more instance, 1..* at least one instance Purple: visits over time Yellow: clinical forms and imaging Green: specific forms at the Basic Data & Diagnosis visit Red: specific forms at closure Grey: Specific forms at any time-point
